# Magnetic resonance imaging features of breast cancer according to intrinsic subtypes: correlations with neoadjuvant chemotherapy effects

**DOI:** 10.1186/2193-1801-3-240

**Published:** 2014-05-09

**Authors:** Hiroko Kawashima, Masafumi Inokuchi, Hiroyuki Furukawa, Hiroko Ikeda, Seiko Kitamura

**Affiliations:** Department of Quantum Medical Technology, Graduate School of Medical Science, Kanazawa University, 5-11-80 Kodatsuno, Kanazawa, 920-0942 Japan; Section of Breast Oncology, Kanazawa University Hospital, 13-1 Takara-machi, Kanazawa, 920-8641 Japan; Division of Pathology, Kanazawa University Hospital, 13-1 Takara-machi, Kanazawa, 920-8641 Japan

**Keywords:** Magnetic resonance imaging, Breast cancer, Intrinsic subtype, Neoadjuvant chemotherapy

## Abstract

**Purpose:**

The purpose of this study was to evaluate the magnetic resonance imaging (MRI) features of breast cancer according to intrinsic subtypes and to investigate whether the MRI and immunohistochemical findings were related to neoadjuvant chemotherapy (NAC) effects.

**Materials and methods:**

The MRI in 116 women with breast cancers who underwent NAC was reviewed. The mass margin, presence of intratumoral necrosis, tumor extension around the mass, relative signal enhancement (RSE), and kinetic curve pattern were analyzed. We investigated the possible correlations between MRI findings and the effects of NAC.

**Results:**

An irregular mass margin was significantly associated with luminal-A cancers, while a smooth mass margin was associated with human epidermal growth factor receptor2 (HER2) cancers. Intratumoral necrosis was significantly associated with triple-negative cancers. Tumor extension around the mass was significantly infrequent in luminal-B cancers and frequent in HER2 cancers. Luminal-B and HER2 cancers showed a significantly higher RSE at 2 min than Luminal-A cancers. Estrogen receptor (ER)-positive cancers, HER2-negative cancers, and presence of intratumoral necrosis were significantly associated with the NAC non-response.

**Conclusions:**

Several MR features can be used to predict the intrinsic subtype of breast cancers. ER-positivity, HER2-negativity, and presence of intratumoral necrosis were significantly associated with NAC non-response.

## Introduction

It has become clear that breast cancers can be divided into biologically different intrinsic subtypes by gene expression analysis (Perou et al. 2000; Sorlie et al. 2001; Perou & Borresen-Dale 2011; Brenton et al. 2005). In clinical practice, it is common to determine the patient’s intrinsic subtype using an immunohistochemical technique (Tamimi et al. 2008), and a therapeutic plan is formed based on each intrinsic subtype. From this trend, diagnosing breast cancer imaging while keeping the intrinsic subtype in mind is becoming a more widespread practice.

Neoadjuvant chemotherapy (NAC) has been the standard treatment for locally-advanced breast cancer (Makhoul & Kiwan 2011). NAC is mainly designed to reduce the tumor size, thereby permitting breast-conserving surgery (Liu et al. 2010). Other advantages of NAC are that systemic therapy may be initiated earlier, and it enables evaluation of a patient’s response to chemotherapy (Kaufmann et al. 2007; Kaufmann et al. 2006). Conversely, a NAC-related disadvantage is the delay of surgical treatment in patients who do not respond to chemotherapy. If we can predict the final chemotherapeutic response during earlier NAC courses, an earlier and optimized treatment regimen is possible, thereby improving patient prognosis (Chollet et al. 2002; Montagna et al. 2010).

The purpose of this study was to evaluate the magnetic resonance imaging (MRI) features of breast cancer according to the intrinsic subtypes, and to investigate whether the MRI and immunohistochemical findings were related to NAC effects.

## Materials and methods

### Patients and treatment

Kanazawa University Medical Ethical Review Board approved this retrospective study, and the requirement for informed consent was waived. We enrolled patients with breast cancer who underwent NAC at our hospital between March 2008 and January 2013. A core biopsy of the tumor was performed prior to NAC to determine the histological diagnosis, hormone receptor status, human epidermal growth factor receptor 2 (HER2) overexpression status, and Ki-67 labeling index. We divided patients into five intrinsic subtypes. The subjects included 116 women (24 luminal A, 15 luminal B, 14 luminal-HER2, 23 HER2, and 40 triple-negative cancers) (Table 1). All cases showed mass formation and the mean maximal tumor size was 28 mm (range, 13-69 mm). The Ki-67 labeling index has not measured for the initial 42 cases; therefore, we judged luminal A or B status according to proliferative parameters such as the nuclear grade.

**Table 1:** **Substitutional definition of intrinsic subtype**

Intrinsic subtype	ER	PgR	HER2	Ki-67
Luminal A	+	+/-	-	<14%
Luminal B	+	+/-	-	≧14%
Luminal-HER2	+	+/-	+	any
HER2	-	-	+	any
Triple-negative	-	-	-	any

*ER* estrogen receptor, *PgR* progesterone receptor, *HER2* human epidermal growth factor receptor 2.

The therapeutic schedule and treatment plan were explained to the patient, and informed consent was obtained from all patients. The standard protocol is four cycles of a combination of 100 mg/m^2^ epirubicin, 500 mg/m^2^ cyclophosphamide, and 500 mg/m^2^ fluorouracil every 3 weeks (FEC4), followed by four cycles of 75 mg/m^2^ doxetaxel every 3 weeks (DOC4).

### Magnetic resonance imaging

MRI was performed on a 3.0 T magnet (Signa HDxt 3.0 T, GE Healthcare, Tokyo, Japan) using an eight-channel breast phased array coil. The patients underwent imaging while in the prone position. Sagittal T1-weighted images were obtained using a spin-echo sequence (TR/TE [repetition time/echo time], 500/7.6 ms; FOV [field of view], 20 cm; section thickness, 4 mm; gap, 1 mm; matrix, 320 × 192; NEX [number of excitation], 2). Sagittal T2-weighted images with fat suppression were obtained using a fast spin-echo sequence (TR/TE, 3834/102 ms; FOV, 20 cm; section thickness, 4 mm; gap, 1 mm; matrix, 288 × 224; NEX, 2; acquisition time, 2 min and 39 s). Axial diffusion-weighted images of both breasts were obtained at b values of 0, 800, and 1500 s/mm^2^ using a single-shot echo-planar sequence (TR/TE, 8500/105 ms; FOV, 36 cm; section thickness, 6 mm; no gap; matrix, 128 × 128; NEX, 5; acquisition time, 4 min and 32 s). A dynamic study of both breasts was obtained in an axial plane. A three-dimensional spoiled gradient recalled acquisition in the steady state sequence (TR/TE/TI [inversion time], 6.0/2.5/18 ms; flip angle, 10°; FOV, 32 cm; section thickness, 1.8 mm; matrix, 352 × 352; acquisition time, 1 min and 5 s) was used. Following the first and second scans in an axial plane after the administration of gadopentetate dimeglumine (0.1 mmol/kg body weight), a third scan in a sagittal plane and a fourth scan in an axial plane were acquired.

### Image analysis

In all cases, MRI was performed a total of three times: prior to NAC, after the first half of NAC, and after the completion of NAC. The chief investigator, a radiologist specializing in breast imaging, performed all breast MRI examinations. MR images were reviewed without knowledge of the clinicopathologic findings. On the MR image prior to NAC, the margin of the mass, presence of intratumoral necrosis, and presence of tumor extension around the mass were analyzed. The margin of the mass was roughly divided into two categories: smooth or irregular. If there was a very high signal intensity (similar to that of water) in the tumor on fat-suppressed T2-weighted MR imaging, we judged it to represent intratumoral necrosis. If there was a similar degree of enhancement around the mass on dynamic MR images, we judged it to represent tumor extension. Dynamic contrast medium uptake was evaluated by relative signal enhancement (RSE) and the kinetic curve pattern. RSE was calculated using the following equation:

The kinetic curve pattern was categorized into three types (washout, plateau, and persistent) according to the BI-RADS MR guidelines (American College of Radiology 2003).

### Histopathologic assessment

A core biopsy of the tumor was performed prior to NAC to determine the histological diagnosis. Immunohistochemical staining for estrogen receptor (ER) and progesterone receptor (PgR), HER2, and Ki-67 was performed. HER2 was evaluated using the HercepTest (Dako, Glostrup, Denmark), and scored on a scale from 0 to 3+. Tumors with scores of ≥3 or with a ≥2.2-fold increase in HER2 gene amplification as determined by fluorescence *in situ* hybridization (FISH) were considered to be positive for HER2 overexpression.

After NAC, all patients underwent either mastectomy or breast-conserving surgery. Surgical specimens from the breast-conserving surgeries were cut into 5-mm slices and those from mastectomies were cut into 5–10 mm slices. The specimens were stained with hematoxylin and eosin for histopathological evaluations.

The pathological response following NAC was evaluated by pathologists according to the Japanese Breast Cancer Society’s criteria for the assessment of therapeutic response (The Japanese Breast Cancer Society 2012). Considering the clinical response, the overall response to chemotherapy was classified into the following five categories: Grade 3: no residual invasive cancer; Grade 2b: only a few remaining cancer cells; Grade 2a: marked changes in two-thirds or more of cancer cells; Grade 1b: marked changes in one-third or more but less than two-thirds of cancer cells; Grade 1a: marked changes in less than one-third of cancer cells.

### Statistical analysis

MRI features according to each intrinsic subtype were analyzed using multivariate logistic regression analysis with the statistical program Stat Flex (version 6, Arthch Inc., Osaka, Japan). To compare the RSE between each intrinsic subtype, we used a multiple comparison method (Dunn’s method). A multiple regression analysis was performed to examine the relationship between the MRI and immunohistochemical findings with NAC effects. A best-fit regression model was sought by use of a stepwise selection method. *P*-values of < 0.05 were considered statistically significant.

## Results

**Figure 1:**
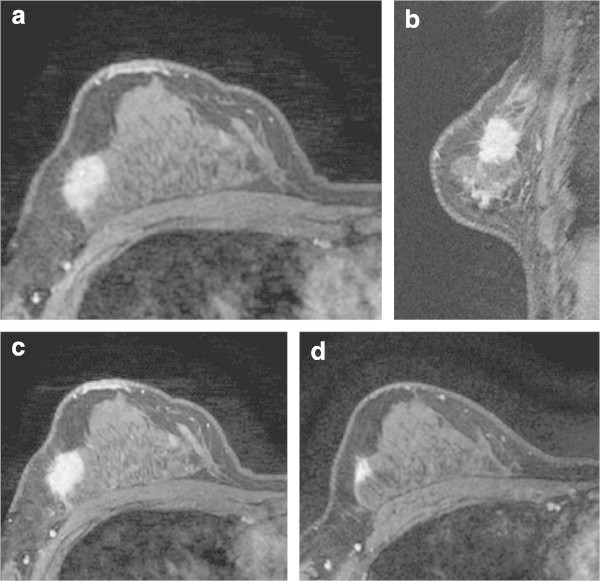
**Luminal A breast cancer of right breast in 40-year-old woman.** Prior to NAC, dynamic MR images obtained at 60 s **(a)**, 240 s **(b)**, and 300 s **(c)** after administration of gadolinium show a persistent enhancing mass with an irregular border. After the completion of NAC, residual tumor still shows an enhancement on dynamic MR image obtained at 60 s **(d)**.

**Figure 2:**
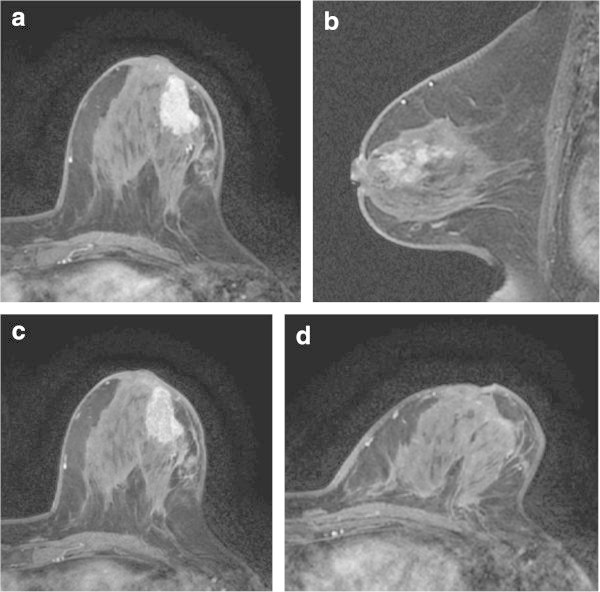
**HER2 breast cancer of left breast in 71-year-old woman.** Prior to NAC, dynamic MR images obtained at 60 s **(a)**, 240 s **(b)**, and 300 s **(c)** after administration of gadolinium show a strong and washout enhancing mass with a smooth border. Tumor extension around the mass is shown in Figure 2b. After the completion of NAC, there is no longer recognizable tumor on dynamic MR image obtained at 300 s **(d)**.

**Figure 3:**
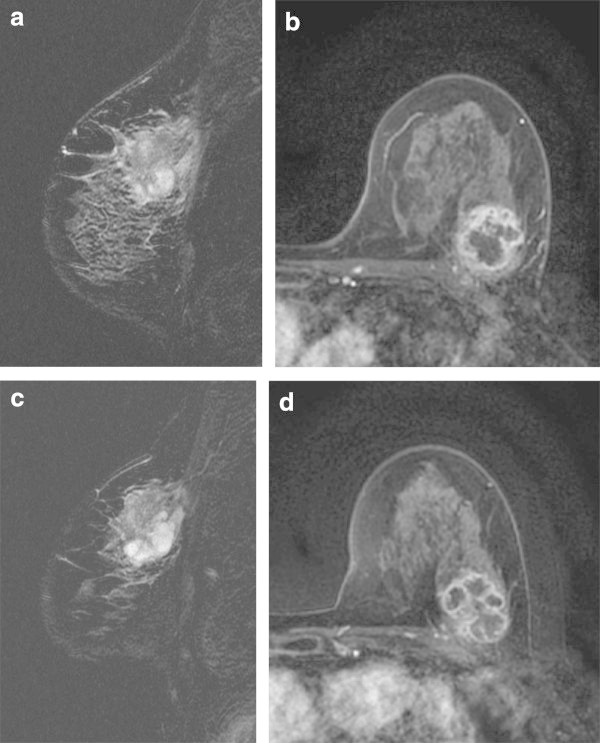
**Triple-negative breast cancer of left breast in 69-year-old woman.** Prior to NAC, fat-suppressed T2-weighted MR image shows a mass with an area of very high intratumoral intensity **(a)** and dynamic MR image obtained at 60 s after administration of gadolinium shows a peripherally-enhancing mass with a large area of intratumoral necrosis **(b)**. After the completion of NAC, very high intratumoral intensity enlarges on fat-suppressed T2-weighted MR image **(c)** and there is an increase in tumor size on dynamic MR image obtained at 60 s **(d)**.

MRI features according to each intrinsic subtype are shown in Table 2. An irregular mass margin was significantly associated with luminal A cancers (P =0.005) (Figure 1) and a smooth mass margin with HER2 cancers (P =0.032) (Figure 2). Intratumoral necrosis was significantly associated with triple-negative cancers (P =0.019) (Figure 3). Tumor extension around the mass was significantly infrequent in luminal B cancers (P =0.0047) and frequent in HER2 cancers (P =0.009) (Figure 2). No significant characteristics were demonstrated by luminal-HER2 cancers. As for the kinetic curve pattern, luminal A cancers frequently showed a persistent pattern, while luminal B and HER2 cancers frequently showed washout patterns. However, the results were not statistically significant.

**Table 2:** **MRI features according to each intrinsic subtype**

	LA	n-LA	LB	n-LB	LH2	n-LH2	H2	n-H2	TN	n-TN
MRI findings	(n = 24)	(n = 92)	(n = 15)	(n = 101)	(n = 14)	(n = 102)	(n = 23)	(n = 93)	(n = 40)	(n = 76)
Mass margin										
Smooth	7(29)	52(57)	7(47)	52(51)	6(43)	53(52)	15(65)^b^	44(47)	24(60)	35(46)
Irregular	17(71)^a^	40(43)	8(53)	49(49)	8(57)	49(48)	8(35)	49(53)	16(40)	41(54)
	^a^P = .005						^b^P = .032			
Intratumoral necrosis										
Presence	4(17)	17(18)	3(20)	18(18)	0(0)	21(21)	2(9)	19(20)	12(30)^c^	9(12)
Absence	20(83)	75(82)	12(80)	83(82)	14(100)	81(79)	21(91)	74(80)	28(70)	67(88)
									^c^P = .019	
Tumor extension around mass										
Presence	17(71)	69(75)	7(47)	79(78)	10(71)	76(75)	21(91)^e^	65(70)	31(78)	55(72)
Absence	7(29)	23(25)	8(53)^d^	22(22)	4(29)	26(25)	2(9)	28(30)	9(22)	21(28)
			^d^P = .0047				^e^P = .009			
Kinetic curve pattern										
Washout	5(21)	37(40)	8(53)	34(34)	5(36)	37(36)	14(61)	28(30)	10(25)	32(42)
Plateau	14(58)	44(48)	6(40)	52(51)	8(57)	50(49)	6(26)	52(56)	24(60)	34(45)
Persistent	5(21)	11(12)	1(7)	15(15)	1(7)	15(15)	3(13)	13(14)	6(15)	10(13)

*LA* luminal A, *n-LA* non-luminal A, *LB* luminal B, *n-LB* non-luminal B, *LH2* luminal-HER2, *n-LH2* non-luminal-HER2, *H2* HER2, *n-H2* non-HER2, *TN* triple-negative, *n-TN* non-triple-negative.

^a,b,c,d,e^Indicates statistical significance (P < .05).

**Figure 4:**
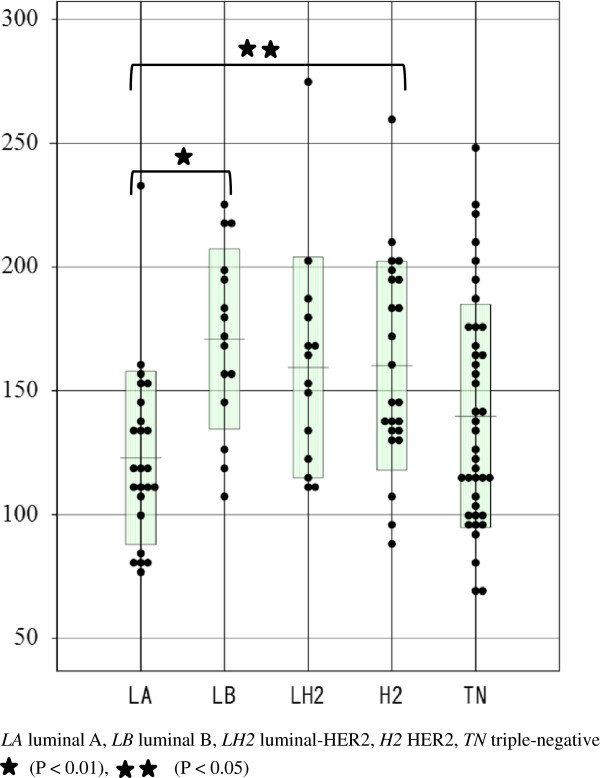
**Relative signal enhancement (RSE) at 2 min according to each intrinsic subtype.** Luminal B cancers showed significantly higher RSE than luminal A cancers (P < 0.01), and HER2 cancers also showed significantly higher RSE than luminal A cancers (P < 0.05).

**Figure 5:**
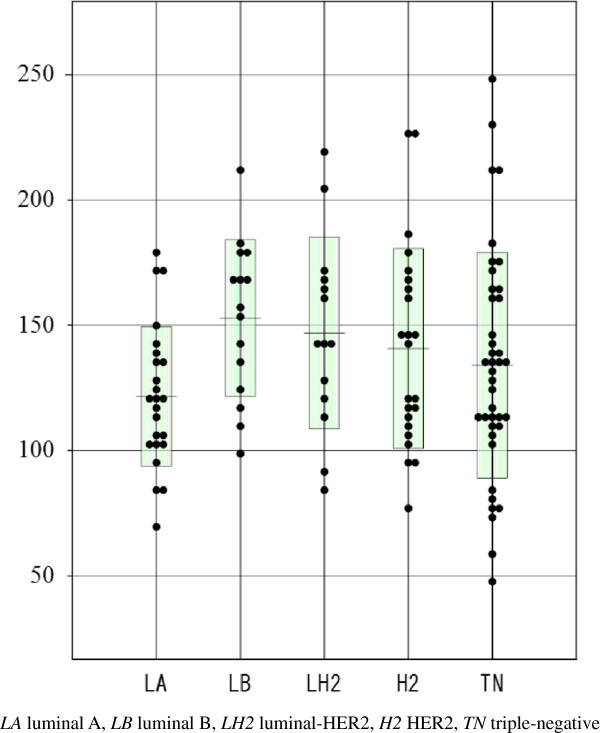
**Relative signal enhancement (RSE) at 6 min according to each intrinsic subtype.** RSE at 6 min was not significant different between each intrinsic subtype.

RSE according to each intrinsic subtype is shown in Figures 4 and 5. Luminal B cancers showed a significantly higher RSE than luminal A cancers (P < 0.01), and HER2 cancers also showed a significantly higher RSE than luminal A cancers (P < 0.05) at 2 min (Figure 4). RSE at 6 min was not significantly different between the intrinsic subtypes (Figure 5).

**Figure 6:**
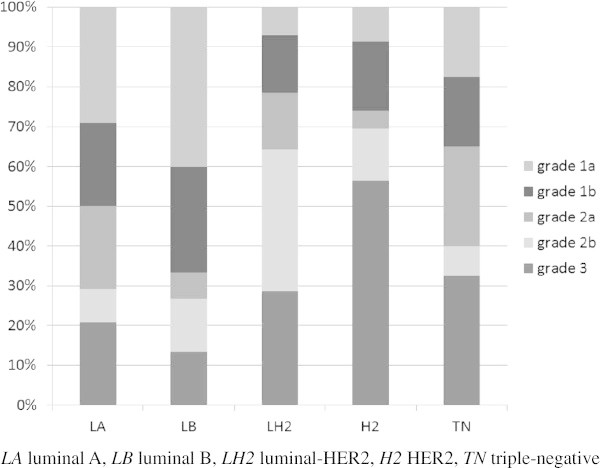
**Pathological response following NAC according to each intrinsic subtype.**

The pathological response following NAC according to each intrinsic subtype is shown in Figure 6. HER2 cancers showed the highest pathological complete response (pCR) rate (Grade 3); on the other hand, luminal B cancers showed the lowest pCR rate. A multiple regression analysis was performed to examine the relationship between the MRI and immunohistochemical findings with NAC effects (Table 3). ER-positive cancers (P =0.024), HER2-negative cancers (P =0.022), and presence of intratumoral necrosis (P =0.016) were significantly associated with NAC non-response. PgR and Ki-67 status, margin of the mass, presence of tumor extension around the mass, RSE, and kinetic curve pattern were not significantly associated with NAC response.

**Table 3:** **Relationship between NAC effects with the MRI and immunohistochemical findings by multiple regression analysis**

	P value	β	stdβ
ER	.0244	-0.6213	-0.2017
PgR	NS		
HER2	.0222	0.6885	0.2091
Ki-67	NS		
Mass margin	NS		
Intratumoral necrosis	.0157	-0.8826	-0.2214
Tumor extension around mass	NS		
Kinetic curve pattern	NS		
RSE 2 min	NS		
RSE 6 min	NS		

*NAC* neoadjuvant chemotherapy, *β* partial regression coefficient, *stdβ* standard partial regression coefficient, *ER* estrogen receptor, *PgR* progesterone receptor, *HER2* human epidermal growth factor receptor 2, *RSE* relative signal enhancement.

## Discussion

The major principles of treatment for breast cancer patients are standardization and individualization. Namely, while employing an evidence-based standard treatment as a foundation, the details of individual treatment protocols are established based on the level of risk of each individual patient. Breast cancer is a heterogeneous disease and it has become clear that breast cancer can be divided into biologically different intrinsic subtypes by gene expression analysis (Perou et al. 2000; Sorlie et al. 2001; Perou & Borresen-Dale 2011; Brenton et al. 2005). Additionally, in clinical care, it is common that the patient’s intrinsic subtype is determined using an immunohistochemical technique (Tamimi et al. 2008), and a therapeutic plan is formed based on each intrinsic subtype. Among the several intrinsic subtypes, triple-negative breast cancer experientially shows characteristic imaging findings. Uematsu *et al.* have reported that MRI findings including a smooth mass margin, rim enhancement, and very high signal intensity on T2-weighted imaging were significantly associated with triple-negative breast cancers compared to ER-positive, PR-positive, and HER2-negative cancers (Uematsu et al. 2009).

Recently, some reports have shown the evaluation of the MRI findings of all breast cancer subtypes; however, few characteristic findings have been revealed, except for triple-negative breast cancer (Hao et al. 2013; Bae et al. 2014; Yamaguchi et al. 2014). The results of our study show that an irregular mass margin was significantly associated with luminal A cancers. Luminal A cancers are generally slow-growing and histologically rich in fibrous components. These characteristics are thought to be a cause of the irregular mass margin. The tendency toward a kinetic persistent pattern and low RSE at 2 min in luminal A cancers are also speculated to originate from the same reason.

In our study, a smooth mass margin and tumor extension around the mass were significantly associated with HER2 cancers. The tendency toward a kinetic washout pattern and high RSE at 2 min in HER2 cancers are thought to reflect the highly proliferative activity of HER2 cancers. Luminal B cancers also showed a tendency toward a kinetic washout pattern and a high RSE at 2 min; however, tumor extension around the mass was significantly infrequent, in contrast to HER2 cancer. As no significant characteristics were demonstrated by luminal-HER2 cancers, further analysis concerning the differentiation of luminal B and luminal-HER2 cancers is required.

Kawashima *et al.* reported a variety of imaging findings in triple-negative cancers (Kawashima et al. 2011). In the present study, intratumoral necrosis was significantly associated with triple-negative cancers; however, other imaging findings were not significantly associated. Further fragmentation of triple-negative cancers by gene expression analysis has already commenced; therefore, the methods of imaging analysis are expected to change in the near future.

As NAC becomes more widespread, there have been more opportunities to conduct evaluations via diagnostic imaging. In particular, MRI plays a central role (Rosen et al. 2003; Partridge et al. 2002; Londero et al. 2004; Balu-Maestro et al. 2002; Akazawa et al. 2006). MRI has been proposed to have a role in early response assessment after initiating NAC (Abraham et al. 1996; Yeh et al. 2005). Contrast-enhanced MRI is known to enable an accurate assessment of tumor response after NAC (Heldahl et al. 2011; Liu et al. 2011), and diffusion-weighted MRI is also helpful in predicting response to NAC (Theilmann et al. 2004; Thoeny & Ross 2010). Recently, there have been reports on predictions of treatment effects made by focusing on the kinetic parameters of dynamic MRI and predictions of effects using parametric MRI (Abramson et al. 2013; Jafri et al. 2013; Koo et al. 2012).

The former investigator reported differences in pre-treatment imaging findings between responders and non-responders in triple-negative cancers and found that no effective treatment response can be expected in cases with an irregular mass shape and intratumoral necrosis (Kawashima et al. 2011). In the present study, we investigated the correlation between pre-treatment MRI findings and final NAC effect for all breast cancer subtypes. From the viewpoint of immunohistochemical status, ER-positive and HER2-negative cancers were significantly associated with NAC non-response. This result is compatible with our clinical experience. Ki-67 status was not significantly associated with NAC response. This result might be caused by the small number of cases have measured the Ki-67 labeling index.

Among the MRI findings, only the presence of intratumoral necrosis was significantly associated with NAC non-response. The margin of the mass, presence of tumor extension around the mass, RSE, and kinetic curve pattern were not significantly associated with NAC response. These results might be caused by the relatively small number of luminal B and luminal-HER2 cancers in the present study compared to the other subtypes.

Our study has several limitations. First, we enrolled patients who were NAC-recipients and who underwent MRI on the same 3.0 T machine. Therefore, there may be a bias due to selecting NAC cases and the number of cases is insufficient. Further studies with larger numbers of cases are needed. Second, the Ki-67 labeling index was not measured in patients in the early period of the study. Third, the presence of tumor extension around the mass on MRI was not confirmed pathologically. While no definitive conclusions can be made from the present study because of these limitations, a variety of MRI findings was shown, even within each intrinsic subtype. Further detailed analysis of MRI findings in each intrinsic subtype in parallel with the fragmentation of each intrinsic subtype by gene expression analysis is expected.

In conclusion, several MR imaging features might be used to predict the intrinsic subtype of breast cancer. ER-positive cancers, HER2-negative cancers, and the presence of intratumoral necrosis were significantly associated with the NAC non-response.
